# Auditory hallucinations and migraine of possible brainstem origin

**DOI:** 10.1007/s10194-011-0355-z

**Published:** 2011-06-04

**Authors:** Y. L. Lo, S. Hameed, H. Rumpel, L. L. Chan

**Affiliations:** 1 Department of Neurology, National Neuroscience Institute, Singapore General Hospital, Outram Road, Singapore, 169608 Singapore; 2 Department of Diagnostic Radiology, Singapore General Hospital, Outram Road, Singapore, 169608 Singapore

## Abstract

**Background:**

Concurrence of migraine and hallucinations is extremely rare and the underlying mechanism is poorly understood.

**Methods:**

We report a 22-year-old man with migraine associated with auditory hallucinations. Concurrent psychotic illness has been excluded.

**Results:**

Brain MR scans showed a stable, patchy FLAIR hyperintensity over the posterolateral aspect of the left cerebral peduncle, just below the level of the red nucleus. This was felt to represent an area of gliosis based on the interval stability over 19 months. There was absence of features for aggressive neoplasms, such as lesional high cellular turnover (choline/NAA ratio >1.0) or high cerebral blood volume on advanced MR imaging with MR spectroscopy and dynamic perfusion MR. EEG and brainstem auditory evoked potentials were unremarkable.

**Conclusions:**

To our knowledge, there are no reports to date on similar auditory hallucinations in adult migraine patients, as well as with associated MRI brainstem lesions. The peduncular lesion could represent a previous migrainous infarct, and a possible analogy can be drawn from the descriptions of peduncular hallucinosis. Brainstem lesions, particularly in the midbrain and pons, have rarely been associated with this condition. It has been postulated that the damage to ascending reticular systems or thalamocortical circuitry may contribute to its pathogenesis.

## Introduction

Concurrence of migraine and hallucinations is extremely rare. The underlying mechanism is poorly understood, but disturbances in regional blood flow [1] may be a contributory factor. We describe a case of distinct, non-psychotic auditory hallucinations in a patient with a brainstem lesion seen on MR scans.

## Case report

A 22-year-old man had presented with recurrent pulsating right frontal headaches of moderate severity. The attacks occur two times weekly, lasting up to 6 h each, often with nausea. The headache episodes were associated with non-vertiginous giddiness and photophobia. He noticed that the strong odors consistently triggered an attack. No family history of migraine or psychiatric disease was documented.

He complained of hearing “people talking or shouting” preceding each episode of headache, lasting up to an hour in duration. The auditory phenomena were distinctly “of several individuals having a conversation”, but “conversations heard clearly did not involve the patient himself”. There were no reports of buzzing or ringing typical of tinnitus. Although these hallucinations did not occur strictly with each attack, they were frequent enough for him to seek medical consultation. He, however, did not experience hallucinations without headache episodes. No visual disturbances were reported during this period. Psychiatric evaluation was performed and excluded thought disorder.

He was prescribed propanolol daily but experienced partial improvement. Addition of amitryptyline resulted in almost complete abolition of headaches, as well as the associated auditory hallucinations.

**Fig. 1:**
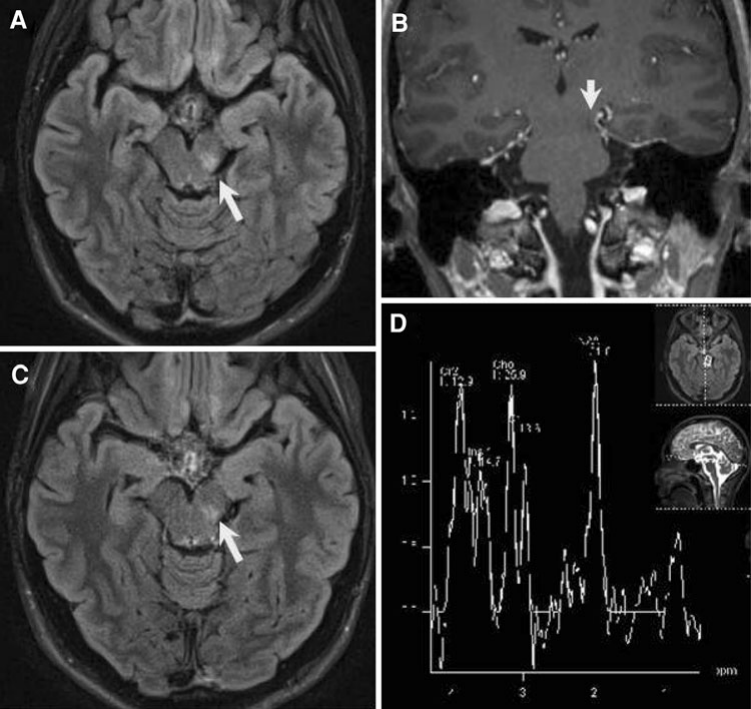
Axial FLAIR (fluid attenuated inversion recovery) (**a**) and coronal post-contrast T1-weighted (**b**) MR image through the midbrain at presentation shows a patchy hyperintense (**a**) and non-enhancing hypointense (**b**) lesion over the posterolateral aspect of the left cerebral peduncle (*arrow*), just below the level of the red nucleus. Follow-up MR (**c**) over 19 months showed a stable lesion (*arrow*). Single voxel H1 MR spectroscopy (**d**), epicentered over the lesion showed a choline/NAA ratio of 0.81

Electroencephalographic (EEG) examination during a headache episode did not reveal any abnormality, as were brainstem auditory evoked potentials. Blood tests, including metabolic and vasculitic screen, were unremarkable. Several repeat brain MR scans (Fig. 1) showed a stable, patchy FLAIR hyperintensity over the posterolateral aspect of the left cerebral peduncle, just below the level of the red nucleus. This was felt to represent an area of gliosis based on interval stability over 19 months. In addition, there was absence of sinister features for aggressive neoplasms, such as lesional high cellular turnover (choline/NAA ratio >1.0) or high cerebral blood volume on advanced MR imaging with MR spectroscopy and dynamic perfusion MR.

## Discussion

The patient’s symptoms had not only fulfilled the International Headache Society classification for migraine [2], but also included an unusual feature of auditory hallucination manifesting in close relation to each attack. This clinical description, in the absence of thought disorder, renders the diagnosis of psychotic disorder most unlikely. Furthermore, the auditory hallucinations had resolved in tandem with headache episodes after conventional migraine prophylaxis.

Abnormal perceptual experiences reported in migraine include visual, olfactory, gustatory hallucinations and distortion of body image [3, 4]. Auditory hallucinations described have occurred exclusively in children, usually consisting of repetitive sounds described as “beeping” or “radio playing” noises. Imaging of the brain has been negative for these patients. Subjectively, they may prove difficult to distinguish from tinnitus which is also well known to occur in migraine [5]. To our knowledge, there are no reports to date on similar auditory hallucinations in the adult age group.

The possible role of the peduncular MRI abnormality as an explanation for the patient’s auditory symptoms needs to be considered. The lesion is situated along the lateral lemniscus, where auditory fibers ascend from the cochlear nuclei to the inferior colliculus. In addition, neuroimaging has demonstrated the posterior circulation as being most vulnerable for ischemic strokes in migraine, although the underlying reason for this distribution is unclear [6]. In this patient, the peduncular lesion could represent a previous migrainous infarct as a result of vasoconstriction [1]. A possible analogy can be drawn from the descriptions of peduncular hallucinosis, although they largely present with visual hallucinations. Brainstem lesions, particularly in the midbrain and pons, have rarely been associated with this condition. It has been postulated that damage to the ascending reticular systems or thalamocortical circuitry may contribute to its pathogenesis [7].

The association of peduncular lesions with auditory hallucinations has previously been reported. These have included midbrain or thalamic infarcts [7, 8], pontine tegmentum lesions [9] and pontine hemorrhage after surgery [10]. Nevertheless, the possibility of auditory aura manifested as auditory hallucination in this patient appears highly unusual and may occur even without the presence of the brainstem lesion. This is particularly so, especially when visual hallucinations are well described in migraine patients [11, 12], in cases where MRI did not show the presence of brainstem lesions. The hallucinations in this patient, as stated, had preceded each attack of headache. However, this sequence of events would not ascertain with certainty that an infarct was the underlying etiology, as each particular hallucination/headache episode would certainly not result in an infarct indefinitely. Rather, it has been postulated that the loss of brainstem control of descending cortical processes [7] may be involved in the pathogenesis of such hallucinations.

This most unusual case provides a plausible relation between migraine, auditory hallucinations and brain stem pathology. As this is an extremely rare finding, future clinical observation will better define if these are merely chance occurrences.

Informed consent obtained.
